# Role of public relations practices in content management: the mediating role of new media platforms

**DOI:** 10.3389/fsoc.2023.1273371

**Published:** 2024-02-02

**Authors:** Ali Yahya Al Hadeed, Ihsan Maysari, Mohammad Mahmoud Aldroubi, Razaz Waheeb Attar, Farhan Al Olaimat, Mohammed Habes

**Affiliations:** ^1^Public Relations and Advertising Department, Yarmouk University, Irbid, Jordan; ^2^Emirates News Agency, Abu Dhabi, United Arab Emirates; ^3^Department of Arabic Language, Faculty of Art and Humanities, Al al-Bayt University, Mafraq, Jordan; ^4^Department of Business Administration, College of Business and Administration, Princess Nourah Bint Abdulrahman University, Riyadh, Saudi Arabia; ^5^Radio and TV Department, Yarmouk University, Irbid, Jordan

**Keywords:** public relations, new media, two-way communication, audience, content management, media organizations

## Abstract

Public relations practices are widely accompanied by communication and persuasion. Especially today, when new media platforms provide direct accessibility, communication through PR has become more improved. This research focused on media organizations in the UAE, with a special consideration given to their audience content management. The researchers applied the case study method and selected a sample of *n* = 280 individuals from *n* = 12 media houses currently working in the UAE. The results obtained by structural equation modeling (SEM) revealed that media organizations in the UAE pay significant consideration to public relations practices (*p* > 0.000) and new media adoption (*p* > 0.000). Moreover, both these public relations practices (*p* > 0.000) and new media adoption were also found to significantly focus on two-way communication. Consequently, this two-way communication is significantly affecting content management among these organizations (*p* > 0.000), leading to the design, evaluation, and alteration of content that is acceptable and liked by their audiences. Thus, it has been concluded that media content and its management is not a simple task. Audience and communication are two basic factors that play an important role in this regard. Furthermore, the role of public relations practices also enhances communication and content management practices, leading to even more constructive outcomes.

## 1 Introduction

Public relations practices are keenly focused on creating and sustaining relationships with customers and clients through communication processes. This communication is positive, and constructive and contains persuasive tactics that are appealing to listeners (Ahmad and Benazirabad, 2018). Notably, Edwards and Pieczka (2018) consider public relations experts to be creative and emotionally intelligent in different ways. The purpose is not only to communicate and send messages to their listeners but also to acquire favorable feedback from them. As a result, public relations experts in different organizations are considered highly important, having different functions with client-focused approaches (Qruni and Qrunig, 2017). Bashir and Aldaihani (2017) further described the function of public relations experts in a media organization as helping to maintain, foster, and improve relations with other organizations and audience members. PR practices in media organizations are focused on keeping an eye on the current trends being followed by other organizations and what the audience wants to see, read, and follow. All these functions are made possible through PR practices that are enriched with the communication process. As argued by Tworzydło et al. (2020), communication is the key process to increase understanding and achieve goals accordingly. As a result, when public relations practices are focused on audiences, they significantly foster the value of the media content. However, while researchers and critics sometimes differentiate between public relations and communication, they are intertwined and work hand in hand (Guidry et al., 2017). Sometimes, public relations also works as a one-way channel, especially when the information or content is imposed, and the focus is to have a positive public image. Nonetheless, in media organizations, public relations practices are mostly two-way, equally prioritizing audience feedback to further proceed with the production and dissemination of likeable media content (Gimaliev et al., 2020).

Similarly, public relations practices are significantly updated today (Gallicano et al., 2021). According to Savič (2016), the more organizations are focused on communication, the more they are likely to improve their PR practices. Thus, they perform more strategically and effectively to meet their goals (Al Olaimat et al., 2022). More specifically, media organizations across the globe are searching for and adopting new approaches to enhance their practices. Here, Michael (2021) cited an example of new media platforms being more effective, direct, and efficient in accessing audiences and obtaining their feedback. As noted, public relations scholars, experts, and critics have widely agreed that new media has changed the landscape of public relations practices (Elareshi et al., 2022). In addition to their ease of use, accessibility, and usefulness, new media platforms are justified resources to be used for public relations practices. In this regard, the empirical evidence on new media in public relations also attests to its role in transforming organizational functioning and content production systems (Rashid et al., 2019; Ureña et al., 2019; Radwan et al., 2021; Singh Apte and Upadhyay, 2022). As noted by Abdalli and Hassan (2019), the role of new media in public relations practices cannot be underscored or denied. Today, many media organizations rely on digital resources for their public relations practices. This dependency indicates several reasons that accelerate new media usage for PR purposes. Particularly, this trend seems more followed by media organizations, where content production and dissemination primarily depend on audience response and feedback (Ahmad and Benazirabad, 2018). To establish a better understanding of the subject of new media, it is important to explore the impact of digital platforms on public relations practices in content management and the advances in social media research. Subsequently, the current study highlights the impact and promise of new technologies and aims to understand how the digital transition can be harnessed to develop and enhance local public relations practices. Similarly, the study of the advances in public relations research has identified multiple emergent themes in the existing corpus, thereby furthering our understanding of advances in new media platforms (Dacko-Pikiewicz, 2021).

### 1.1 Study aims and objectives

It is notable that public relations has long been addressed by researchers in the UAE. However, their focus remained on other factors such as issues in PR practices (Al-Jenaibi, 2015), PR in crisis management (Kamil, 2020), and PR practices in the Emirati banking sector (Muhammad et al., 2019), among others. Yet no study has examined PR in media content management, indicating a primary empirical gap. Thus, to fill this existing gap, this study focused on examining the role of new media platforms in the media content management process. No study has examined public relations in media content management in the Emirati context; rather, most studies have dealt with reputation management and the effects of multimedia in the Arab region, specifically in the Emirates, but they provide valuable insights into the communications landscape and the role of social media in shaping public opinion and guiding consumer behavior. Therefore, in the current study, the researchers focused on public relations practices experts in Emirati media houses who communicate through new media to manage their content according to their audience needs and demands. To provide systematic and empirical evidence of the phenomenon under study, the researchers divided this research into six different sections as the purpose was to provide systematic and empirical evidence. The first section involves an introduction to the topic, problem of the study, objectives, and a brief overview of mass media in the United Arab Emirates. The second section involves a review of the literature, and the third section involves the theoretical support that further assisted in designing the conceptual model of the current research. The fourth section involves the methodological approaches, and the fifth section involves the data analysis and findings of the current study. Finally, in the sixth section, the researchers have discussed the results, and conclusions are made.

### 1.2 Media in the United Arab Emirates

After the British occupation ended in 1971, the mass media landscape in the United Arab Emirates started to evolve. Since then, media platforms have been working under governmental control, being equally controlled and regulated by the National Media Council. Today, there are a total of 19 newspaper organizations, out of which 7 are published in Arabic and the remaining 12 in English (Addi et al., 2021). Furthermore, there are 14 independent radio stations also working in the country, and the rest are government owned. Regarding television, the first TV station in the United Arab Emirates, called “Abu Dhabi Television,” was established in 1969, which led to the beginning of the television era in the 1990s (Aleisaei, 2019). However, currently, the trend of chain ownership is also being followed by the Emirati media organizations. The major media organizations include the MBC Group (UAE), Arab Media Group, Dubai Media Incorporated, Sharjah Media Corporation, and Abu Dhabi Media Incorporated. Dubai Media Incorporated, also known as Emirati Media Incorporated, is owned by the government (Del Chiappa, 2017). It is also notable that these media organizations acknowledge online platforms as equally important for their visibility, content production, dissemination, and interaction with their audiences. As a result, when the COVID-19 outbreak emergency happened, Emirati mass media platforms not only worked to send their content through conventional resources but also resorted to new media platforms for content dissemination and interaction purposes (Myrzabekova, 2021). The UAE is witnessing continuous development in new media as the government seeks to develop national media content to keep pace with developments and changes in the media landscape. These developments include the use of artificial intelligence and future new media in creating media and technological content (Radwan et al., 2021). The UAE is considered a leading destination for media scene makers as it hosts many major media companies and international media forums. New forms of media in the UAE include social media and the Internet; indeed, the UAE has a high Internet penetration rate and ranks first in the world in the use of social media. The UAE government is working to develop a comprehensive media system that includes modern mechanisms, tools, and content that keep pace with the developments and changes taking place in the media landscape. The UAE is also witnessing growth in the new media sector, with many radio and television stations that transmit news and information (Radwan et al., 2021).

## 2 Literature review

### 2.1 Online platforms and media content

An online platform is defined as a means of communication through which the user creates an account that enables them to communicate via the Internet with other people electronically. Online platforms and social networking include many different applications and tools (Wang et al., 2023) that are formed into Internet apps, which are classified as social media. Social media technology takes many forms, including blogging, forums, photo sharing, video blogging, and music and audio sharing via the social networking protocol (Al Jwaniat et al., 2023). It can connect many types of software that enable the use of online platforms and online media content to improve online communication between social media users (Facebook, Tik Tok, YouTube). The term “media content” refers to information and experiences disseminated in specific contexts, via digital and traditional media, for the benefit of end users and audiences in various fields (Tawafak et al., 2023). Media content can include many forms, including newspaper articles, books, video and audio clips, photos, graphics, graphs, charts, animation, games, applications, educational platforms, content management, and public relations. Internet and social media platforms can be used to publish and manage media content and interact with the public (Hatamleh et al., 2023).

### 2.2 Public relations in media organizations

According to Dacko-Pikiewicz (2021), with an abundance of information available through mass media platforms, organizations focus on constant communication with their clients and audiences. In particular, media organizations pay keen attention to their audiences with the aim of creating and sharing content that is mutually acceptable and liked by their audiences. Lingel (2020) considers interaction and communication with the audience as one of the key considerations for media organizations. For this purpose, public relations departments and experts are among the crucial factors that ensure continuous communication with audiences at different levels. Referring more specifically to the role of public relations, Berg and Blomqvist (2019) argued that PR practices are creating a culture of dialogue and communication between organizations and individuals. Earlier, the role of public relations was to inform the public, but today, this role has been transformed and enhanced to communication. The focus on clients and their feedback is comparatively increased. As a result, communication is two-way, where clients are not only listening to what organizations have, but organizations must also listen to what their clients expect from them (Valentini et al., 2018). Although many researchers consider the role of public relations as like that of advertising and promotion, prioritizing audience feedback is an important element that differentiates between PR and advertising in organizations, especially media-based organizations (Navarro et al., 2017). Therefore, media organizations are considered a major means of communicating with the public, and the field of public relations depends greatly on effective communication. The media can be a means of conveying brand or institution messages effectively, and it can be said that there is a close interaction between media organizations and the field of public relations; indeed, media organizations present a powerful means that can support public relations strategies and contribute to achieving their goals (Berg and Blomqvist, 2019).

*H1*: Media organizations have a significant effect on public relations.

### 2.3 Organizations preferring new media

Today, organizations search for new approaches to increase their accessibility to both other organizations and individuals. However, this access depends on the organizational objectives and their business requirements (Radwan et al., 2021). According to Allagui and Breslow (2017), when organizations are client centric, they aim to reach the masses, interact with them, and gather their feedback to improve existing services and adopt new ones according to their needs and demands. For this purpose, Toledano and Avidar (2019) cited an example of surveys and older ways of gathering feedback and responses through postal services. In this regard, organizations would reach their clients and gather their responses in handwritten forms. However, today, trends have changed due to new media resources and platforms.

According to Lee et al. (2015), new media have tremendously increased access to clients and consumers. Traditional methods of response gathering are widely altered as organizations prefer virtual digital platforms to communicate. This is further validated by Valentini et al. (2018), who consider new media as facilitating maximum communication and interactivity. These platforms not only facilitate access to the audience but also provide the audience with access to the organizations they want. Here, Habes et al. (2023) cited an example of public awareness and messages sent to the Pakistani audience during the COVID-19 outbreak. According to the researchers, the relevant campaigns were effective yet required audience feedback as to their adoption of the measures to mitigate the virus transmission process. Consequently, the messages being circulated through conventional platforms were also disseminated through new media resources, which further helped to observe the audience behavior as the feedback to the relevant messages, leading officials to improve and even change the strategies as per the requirements (Iqbal and Khan, 2021).

*H2*: Media organizations have a significant effect on new media.

### 2.4 New media in public relations

According to Edwards and Pieczka (2018), every organization has strategies for their public relations. Whether promoting or selling products or promoting an idea, public relations practices are of greater concern to them. If organizations lack effective communication, it means their public relations practices have a weak strategy. As noted by Bashir and Aldaihani (2017), a strong public relations plan that is built with close attention to clients’ details is more likely to be strategically organized. However, Gimaliev et al. (2020) have considered a strategically organized plan to be focused on the clients with the aim of gathering maximum feedback and details about their needs, which can be later fulfilled by the improved service quality.

Similarly, Froment et al. (2017) emphasized selecting the right media type for PR practices. In this regard, new media is considered as comparatively more useful, with its potential to obtain and coordinate all the necessary information from both clients’ feedback and observing their behavior online. Hall (2020) has considered new media for public relations as “online media relations” that can be time consuming yet more value to an organization by improving its communication practices. Here, Bergstrom and Poor (2022) cited an example of interaction through Facebook, LinkedIn, Twitter, and other virtual resources by many multinational national organizations. According to Valentini et al. (2018), a crucial difference between digitalized public relations and conventional public relations is all-time availability. New media enhances this availability, and communication is feasible whenever the communicators want it.

*H3*: Public relations has a significant effect on new media.

### 2.5 Two-way communication in public relations

The two-symmetrical model of communication and public relations provide theoretical support to the current research. According to Matthee (Matthee, 2011), two-way symmetrical model is considered as one of the most ethical and practical approach of communication today (Kent et al., 2020). As noted earlier, communication is an effective way to share and receive information between communicators. Through communication, it is easy to send a message and monitor what others respond about it (Rodriguez, 2016). As noted by Navarro et al. (2017), feedback is an integral part of communication. In particular, when communication is goal oriented and focused on responses, communicators necessarily follow the rules of two-way communication. As noted by Tankosic et al. (2016), two-way communication is key to creating and sustaining great mutual understanding. It is an important communication pattern as it not only builds trust but also improve the free flow of ideas at every level. Here, Toledano and Avidar (2019) cited an example of an advertising company introducing and promoting products to attract audiences. The type of message was focused on attracting the audience’s interest and their buying behavior as the feedback or response to the communication process. Similarly, in terms of public relations practices, Lane (2018) has considered the role of PR practitioners as not only sending the message but also adopting problem-solving approaches. As audiences can have queries or confusions, two-way communication patterns in public relations can help to answer them all in an effective and efficient manner. Toledano and Avidar (2019) further noted that encouraging two-way communication can help an organization to flourish at its maximum. When PR practices bolster two-way communication, external communication with clients and audiences improves, helping an organization to reach its goals. When the audience or clients are more vocal about their needs and demands, an organization can work accordingly and fulfill their expectation in a better possible manner (Michael, 2021).

*H4*: Public relations has a significant effect on two-way communication.

### 2.6 New media and two-way communication

Although humans and language are considered the basic components of communication, this proposition seems upgraded by certain components today. This era is characterized by informing, with the concept of information communication technology (ICT) having transformed the landscape of communication processes (Tankosic et al., 2016). Yuan (2017) has considered ICT to comprise powerful tools of communication where both one-way and two-communication are followed at both the micro and macro levels. In particular, in organizations where public relations practices are focused on two-way communication approaches, the role of technology is of greater significance.

In this regard, Macnamara (2017) has considered new media an important source of improving public relations practices across the globe so that it may flourish. Organizations where public relations experts resort to new media continuously monitor and evaluate a company’s performance through client feedback. According to Ertürk (2019), viewing relations with clients and audiences as a crucial factor is a key management consideration for public relations experts. Moreover, using new media platforms also contributes to a company’s reputation at many levels. Thus, Roberts-Bowman (2017) has considered the presence of new media as an important approach for public relations that helps to maintain a constant voice across different communication platforms and increase the market presence of an organization.

*H5*: New media has a significant effect on two-way communication.

### 2.7 Two-way communication and content management

In discussing the technological transformation and new media as a part of public relations practices, it is important to highlight their role in facilitating communication at every level (Radwan et al., 2021). For example, during political events, media organizations and campaigns creators strongly consider the ongoing trends at the social and political levels. These trends help to brainstorm and highlight not only new campaigning ideas but also what people like and pay attention to Alturas and Oliveira (2019). According to Radwan et al. (2021), media content design, production, and management are all performed when audience feedback is carefully obtained. It is important to design and shape content in accordance with the audiences’ interests. In this regard, the researchers consider media content as designed and managed by resorting to two-way communication patterns.

Although audience feedback is not a new phenomenon, it is of much importance due to new policies and strategies. In particular, when online communication helps to access audiences, content management is feasible with just a single click (Bergstrom and Poor, 2022). According to Froment et al. (2017), despite the communication and media focus on audience engagement, it primarily depends on the type of communication. For example, one-way communication is declarative and mostly undermines an audience’s critical thinking and opinion sharing capabilities. Two-way communication, on the other hand, is considered more motivating, magnifying the importance of audience feedback and increasing an audience’s critical thinking and opinion sharing capabilities (Yuan, 2017). Similar patterns of two-way communication were seen during the SARS outbreak in the Middle Easter. Local governments not only provided information about measures but also conducted several online surveys, monitored social media profiles, and designed healthcare content accordingly (Valentini et al., 2018).

*H6*: Two-way communication has a significant effect on media content management.

## 3 Theoretical framework

### 3.1 New media theories approach

The approach of new media theories lies in understanding and analyzing the impact of technology and modern media on society and culture (Yuan, 2017). According to Matthee (2011), new media theories provide a framework for understanding how technological development affects change in values and culture and help explain how social behavior and cultural interaction develop under technologies. New media theories also provide a framework for understanding how media content spreads through digital media, which contributes to studying its impact on society and culture (Sarwar et al., 2023). Likewise, these theories highlight how digital media influences the formation of individuals’ identities through their participation in digital content and social media. New media theories explain how the economic structure of the media affects the quality and quantity of media content produced and how it is distributed in society (Yuan, 2017). The theories also focus on the role of the consumer as an active actor in the process of consuming media content, which helps in analyzing how the individual shapes their media experiences (Radwan et al., 2021). Thus, the new media theory approach contributes to opening new windows of understanding of the social and cultural transformations that occur as a result of technology and digital media. New media theories also focus on the role of the audience as an active actor and participant in the media process (Pasha et al., 2022). In content management in public relations, audience interaction with content is vital as content is designed and managed in a way that encourages interaction and participation (Radwan et al., 2021).

### 3.2 Two-way symmetrical model of communication and public relations

**Figure 1:**
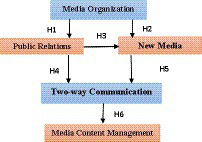
Conceptual model of current research.

The two-way symmetrical model of communication and public relations provides theoretical support to the current research. According to Matthee (2011), the two-way symmetrical model is considered one of the most ethical and practical approaches of communication today. It is focused on communication, which should be built on mutual understanding and an equal opportunity to share and receive the message between both the organization and other parties. As validated by Gruing (2001), the two-way symmetrical model follows a greater social responsibility. An organization following the two-way symmetrical model of communication and public relations strongly prioritizes its workers and audiences to gather their opinion and, thus, work accordingly. In a similar context, this study applies and follows the relevant model, assuming that media organizations in the United Arab Emirates are giving potential importance to communicate with their audiences (Amin et al., 2019). This communication helps them to create and disseminate desirable content that will be accepted by their audiences (feedback). However, compared to the traditional approaches to communicate with audiences, these media houses have their official pages on new media platforms, which are marked by their ease of use and increased accessibility. As a result, greater accessibility to the public facilitates access to their opinion, which further helps them to gather their opinion and shape their content accordingly. Abdalli and Hassan (2019) also consider new media as facilitating communication between PR experts and audiences. As noted, new media not only increases access to audiences but also helps to gather their feedback. Once the audience feedback is gathered, PR experts determine their needs and demands, which further helps organizations in their media content management and production process (Figure 1).

## 4 Research methods

This research involved the case study method. As noted by Crowe et al. (2011), the case study approach is a multifaceted and complex method that helps to assess a phenomenon in a real-life situation. The researchers applied structured questionnaires for the data gathering purposes (Habes et al., 2021b). The scale and items of the questionnaires were adopted from existing research that is summarized in Table 1. Notably, the questionnaire was designed on a five-point Likert scale (Pasha et al., 2021), with *n* = 4 items for each variable. The data gathering was performed from 21st April 2022 to 15th June 2022. After the data gathering (Pasha et al., 2022), the researchers manipulated and coded data for the analysis purposes. As the current research involved structural equation modeling, both Statistical Package for Social Sciences and Amos Ver. 26 were employed.

**Table 1:** Sources of the survey items.

SR	Scales	Sources	No of items
1.	Media organization	Berg and Blomqvist (2019)	04
2.	Public relations	Ahmad and Benazirabad (2018) and Macnamara (2017)	04
3.	New media	Alturas and Oliveira (2019) and Radwan et al. (2021)	04
4.	Two-way communication	Chen et al. (2021) and Rabarison et al. (2017)	04
5.	Media content management	Cunningham et al. (2017) and Fairley and Amherst (2003)	04

### 4.1 Sample selection technique

The population of the current research was composed of public relations practitioners currently working in media houses in the United Arab Emirates. However, as per the research rules, the researchers had to select a subgroup of individuals (Elbasir et al., 2020); thus, the researchers selected *n* = 12 media houses (their regional offices) currently working in the four states of the United Arab Emirates. Furthermore, the researchers selected a sample of *n* = 280 individuals as the study requirements and design. According to Howard (2016), studies that are based on structural equation modeling should contain a minimum sample size of *n* = 200 individuals to ensure the reliability and generalizability of the results. In this regard, the sample size of *n* = 280-was ideal for this research. Finally, the researchers performed data collection by personally visiting the media houses in the selected locations. As the study was focused on public relations practices, the researchers used convenience sampling techniques and only selected PR practitioners operating in the selected organizations. According to Taherdoost (2018), despite convenience sampling having received much criticism from the researchers, it is one of the most preferred sampling methods. However, the relevant techniques help researchers to select and gather data from the most suitable respondents that have a direct and real-life experience of the phenomenon under study (Habes et al., 2021a). Thus, after the data collection process, the researchers collected the questionnaires and carefully checked them before the coding and data analysis. Out of the *n* = 280 questionnaires, *n* = 271 were finalized for the further proceedings, indicating a response rate of 96.7, and *n* = 9 questionnaires were either incompletely filled out or missing.

### 4.2 Ethical considerations

The researchers first took a written permission, signed by the branch managers of the relevant offices regarding the data collection process. Furthermore, the researchers provided the respondents with informed consent as an important consideration suggested by Gov (2013). The respondents were briefed about the research problem, purposes, and the usefulness of the study results. The researchers also assured the respondents that their personal data would be kept confidential and not be used for any personal or commercial purposes.

### 4.3 Common method bias

According to Apuke (2017), common method bias (CMB) occurs when the variation in the gathered data is caused by the research tool instead of the actual responses that it aims to obtain. In simple words, common method bias (CMB) is caused by the tools rather than the data. In this research, the researchers tested the common method bias (CMB) by using Harman’s single factor score (Kock, 2021). The results revealed the total bias at 36.1%, which is smaller than the threshold value of 0.50, indicating that the CMB did not affect the gathered data.

## 5 Analysis and results

### 5.1 Convergent validity

Hussain et al. consider convergent validity as determining the extent to which the scale items are related to other scales of the same construct (Hussain et al., 2018). Precisely speaking, convergent validity provides a pathway to determine the pathway to assess the internal consistency of the measurement model (Nawanir et al., 2018). Examining the internal consistency of the measurement model indicated most of the factor loading as surpassing the threshold value of 0.05. Moreover, the average variance extracted values (0.814–0.923) also remained higher than the relevant threshold value of 0.5. Regarding construct reliability, the analysis revealed the composite reliability values as ranging from 0.761 to 0.829 and the Cronbach alpha values as ranging from 0.754 to 0.873, thus surpassing the threshold value of 0.7. Thus, the convergent validity analysis was established, indicating the questionnaire items as internally consistent (Koonce and Kelly, 2014). Table 2 summarizes the convergent validity calculations.

**Table 2:** Summary of convergent validity analysis.

Constructs	Items	FL	AVE	CA	CR
Media organization	MED1	0.782	0.861	0.756	0.761
	MED2	0.622			
	MED3	0.809			
	MED4	0.992			
Public relations	PR1	−0.164	0.833	0.789	0.788
	PR2	0.885			
	PR3	0.827			
	PR4	0.788			
New media	NMED1	0.809	0.840	0.754	0.801
	NMED2	0.787			
	NMED3	0.852			
	NMED4	0.859			
Two-way communication	COM1	0.869	0.926	0.760	0.799
	COM2	0.920			
	COM3	0.990			
	COM4	0.722			
Content management	MNG1	0.783	0.814	0.873	0.821
	MNG2	0.451			
	MNG3	0.783			
	MNG	0.877			

### 5.2 Discriminant validity

This research further involved discriminant validity to determine whether the research constructs were not highly correlated. As the constructs were weakly correlated, the higher the discriminant validity therefore was (Samuels, 2016). The researchers further examined the discriminant validity by applying the standard two-criterion approach including the heterotrait–monotrait (HTMT) ratio and Fornell–Larcker criterion (Howard, 2016). The researchers first utilized the HTMT ratio and manually calculated it. The results revealed the HTMT value at 0.081, which is lower than the threshold value of 0.90 suggested by Henseler et al. (2015). Furthermore, the Fornell–Larcker criterion revealed that all the squares of the average variance exacted values were greater (0.662–0.857) and had a relatively weaker correlation with each other (Wang et al., 2015). Thus, the findings indicated that the discriminant validity of measurement model was affirmed (see Tables 3, 4).

**Table 3:** Heterotrait–monotrait ratio scale.

	MED	PR	NMD	COM	MNG
MED					
PR	−0.086				
NMD	−0.313	−0.137			
COM	−0.057	0.069	0.061		
MNG	−0.465	−0.067	−0.304	−0.276	

MED, media organization; PR, public relations; NMD, new media; COM, two-way communication; MNG, content management.

**Table 4:** Fornell–Larcker criterion.

	MED	PR	NMD	COM	MNG
MED	**0.741**				
PR	0.264	**0.693**			
NMD	0.591	0.284	**0.705**		
COM	0.271	0.026	0.173	**0.857**	
MNG	0.677	0.248	0.584	0.371	**0.662**

MED, media organization; PR, public relations; NMD, new media; COM, two-way communication; MNG, content management.Bold indicates the term Media.

### 5.3 Goodness of fit

**Figure 2:**
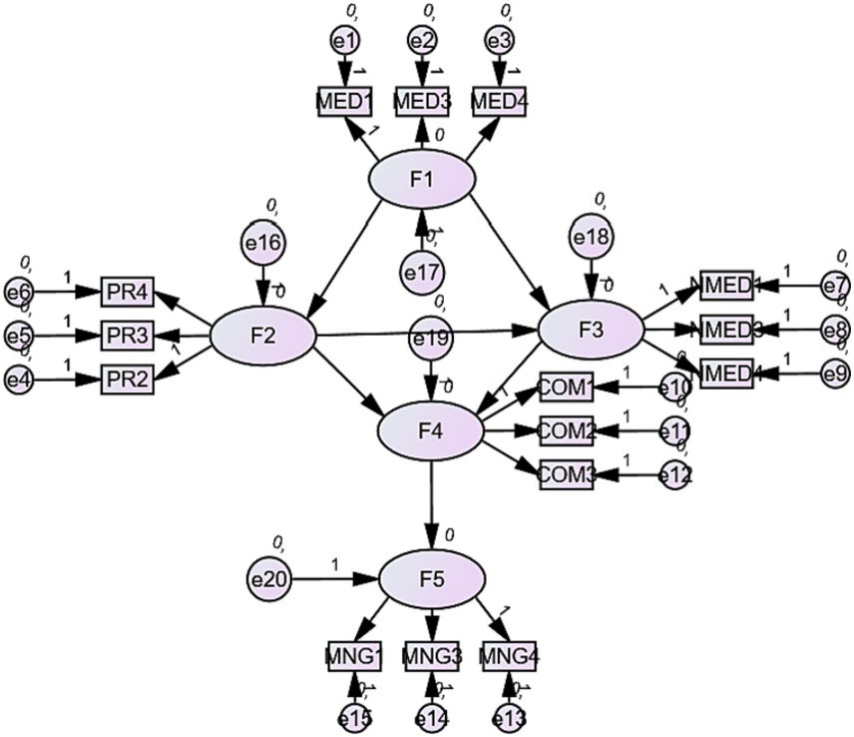
Goodness of fit.

Narsky considers goodness of fit an important component of measurement model analysis in structural equation modeling (SEM) as it determines the extent to which the observed data fit with the expected data under the study model (Narsky, 2004). The goodness of fit in this study revealed the Chi-square value at *x*^2^ = 0.066 (10) and probability value at 0.002. Moreover, the non-fit indices value remained at 0.201 and standardized root mean square (RMSEA) value at 0.370, thus lower than the threshold value of 0.085, indicating that the observed data fit the expected observations with the normal distributions. Figure 2 illustrates the goodness of fit.

### 5.4 *R^2^* analysis of variance

*R^2^* analysis, also known as coefficients of determination *R^2^*, determines the extent to which the exogenous variables can predict the research outcomes (Figueiredo Filho et al., 2011). Primarily, it is based on a number between 0 and 1, indicating the predictive power of the independent construct(s) (Dastres and Soori, 2021). *R^2^* analysis in the current study (see Table 5) revealed 41.1% variance in media organization, 52.9% in new media, 50.1% in two-way communication, and 29.9% variance in content management. Thus, variance ranged from moderate to strong among the latent variables.

**Table 5:** *R*^2^ analysis of the endogenous variables.

Variables	*R^2^*	Strength
Media organization	0.411	Strong
New media	0.529	Strong
Two-way communication	0.501	Strong
Content management	0.299	Moderate

### 5.5 Hypotheses testing

Finally, the researchers examined the causal relationships between the proposed study variables by using the path analysis in the structural equation modeling (Mazouz, 2019). Although regression analysis can also determine the strength and nature of relationships between variables, path analysis provides relatively more details about the paths of relationships in a better possible way (Hair et al., 2021). Thus, the path analysis in this study also contained path values, regression weights, and significance values (see Table 6). Consequently, it was revealed that the effect of media organizations on public relations remained significant, with the path value at 0.188 and significance value at *p* > 0.000. The validation of *H1* indicates its consistency with the study conducted by Toledano and Avidar (2019), who consider media organizations as actively incorporating public relations and communication practices as a part of their strategic functioning and development. Furthermore, *H2* of the current research was focused on the significant effect of media organizations on new media. The relevant hypothesis was adopted from the notion that media organizations are focusing on their new media usage and presence. These organizations use new media to access the public and interact with them for different purposes (Valentini et al., 2018). Analysis revealed that the relevant hypothesis is valid, with the path value at 0.132 and significance value at *p* > 0.006.

**Table 6:** Path analysis of study hypotheses.

Hypotheses	Relationships	Path	*t*	*p*
*H1*.	Media organizations ➔ Public relations	0.188	4.502	***
*H2*.	Media Organizations ➔ New media	0.132	2.747	0.006
*H3*.	Public relations➔ New media	0.377	11.032	***
*H4*.	Public relations➔ Two-way communication	−0.053	−0.408	0.683
*H5*.	New media➔ Two-way communication	0.388	2.879	0.004
*H6*.	Two-way communication➔ Content management	0.251	6.565	***

Furthermore, *H3* in this research proposed a significant effect of public relations and two-way communication. The relevant hypothesis is based on the primary assumption about the role of public relations in creating and sustaining communication under the symmetric model of communication and PR (Tankosic et al., 2016). However, *H4* remained insignificant, with the path value at −0.053 and significance value at *p* > 0.683, indicating that the results remained inconsistent with assumptions where PR practices in Emirati media houses follow two-way communication (Roberts-Bowman, 2017).

Furthermore, the effect of new media on two-way communication (*H5*) was consistent with the fact that existing literature on new media and communication considers it as facilitating two-way interactivity among users (Sembor, 2017). The results revealed the path value at 0.388 and significance value at *p* > 0.004, indicating compatibility with the proposition regarding new media for two-way communication. Finally, the researchers assumed a significant effect of two-way communication on media content management (*H6*). As noted by Aydin et al. (2021), interaction with audience is an important way to design, choose, and display the desirable media content. Organizations that focus on interaction with the audience are more likely to generate suitable content, therefore leading to improved reputation and acceptance among the public. Thus, the analysis revealed the path value at 0.251 and significance value at *p* > 0.000, indicating consistency with the arguments of Ahmad et al. (2019).

### 5.6 Importance performance map analysis

**Figure 3:**
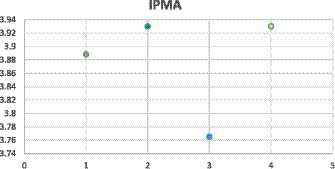
Importance performance map analysis (IPMA).

Importance performance map analysis (IPMA) is an additional step in structural equation modelling that provides an overview of the causal relationships between study variables (Aprilia et al., 2022). IPMA graphically illustrates the performance of latent variables in a graphical form, also known as a “map” (Henseler et al., 2015). Importance performance map analysis (IPMA) in the current research (see Figure 3) indicated public relations (PR) as the highest scoring variable (3.929), while content management (MNG) remained the second highest scoring variable (M 3.921). Followed by media organization (3.88), two-way communication remained the lowest scoring variable.

## 6 Discussion

**Figure 4:**
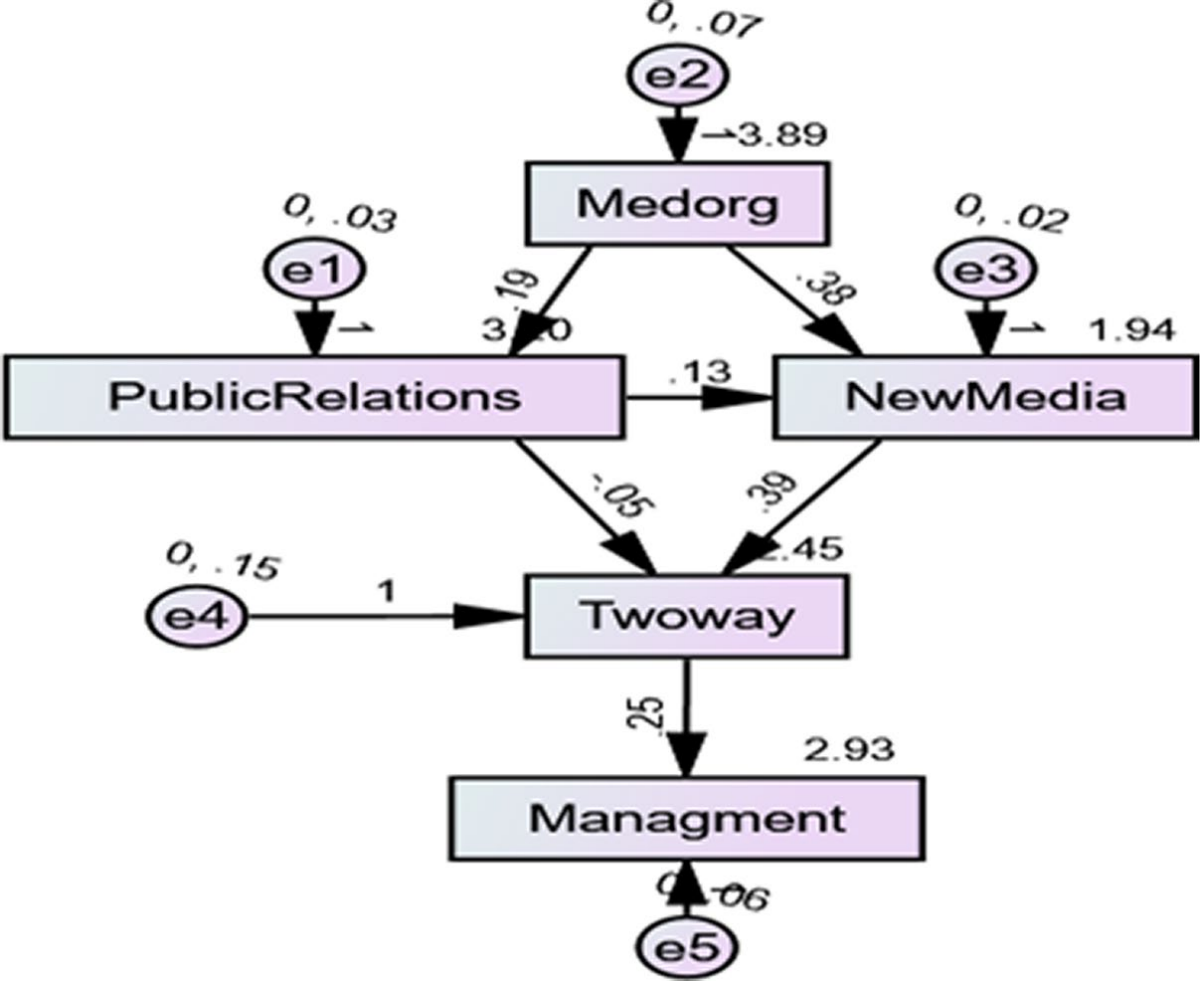
Path coefficient of the model (significant at p** < = 0.01, p* < 0.05).

The two-way symmetrical model of communication proposed by James Gruing’s Excellence Theory aims to examine organizations as focusing on attaining mutual benefits. In other words, this model implies that organizations ensure mutual benefits for themselves and their audiences (Valentini et al., 2018). The basic conceptualization of the Gruing model relies on communication as a primary negotiation between the organization and their public, further fostering mutual understanding and interest (Ahmad and Benazirabad, 2018). A similar approach has been further modified in terms of new media providing different platforms through which people can voice their opinions and organizations can obtain maximum benefit from their feedback (Rahi and Ghani, 2017). According to Aleisaei (2019), the two-way model of communication shows a greater consistency with the propositions about the role of new media in audience feedback and content management. Despite the role of new media, in general terms, remaining prominent, its relevance with PR practices for content management is debatable in many regions, including the United Arab Emirates. As noted by Yuan (2019), the two-way model of communication is very different from the stereotypical perception of the role of public relations of persuading by using deceptive, one-way practices. Rather, public relations experts ensure transparency and equal communication opportunities for all, which further indicates the relevance and significance of two-way communication among them (Tankosic et al., 2016). Public relations practitioners also believe that organizations, individuals, and the public should use communication to modify behavior instead of controlling how others should think and behave (Lee et al., 2015; Figure 4).

Similarly, the focus of this study also remained on public relations practices among Emirati media organizations. Although there are both private and public sector media organizations, the researchers selected the relatively suitable ones to assess their content management. Notably, the first theme of the questionnaire was followed by further root questions, overall investigating the communication patterns and approaches used by the relevant media organizations (see Table 7 for descriptives). Our respondents revealed a democratic communication approach that further helps PR professionals to boost their confidence and provide them with more opportunities to think of and apply creative communication practices (Berg and Blomqvist, 2019). Regarding the second theme and root questions, the focus remained on examining the respondents’ answers about their organizations and public relations practices. The respondents widely agreed with the fact that their organization put a social focus on public relations practices and continuously apply, evaluate, and monitor their PR practices (Abdelrahman Alawaad, 2021). The respondents also addressed and agreed with their organizations’ preferences for new media platforms as providing effective, direct, and efficient communication opportunities. These services, as they indicated, are widely accompanied by ease of access and useful outcomes that further accelerate new media adoption among their organizations (Alghizzawi et al., 2023).

**Table 7:** Descriptives of study responses.

Constructs	Statistics	Descriptives	95% Confidence interval	
			Lower	Upper
Media organization	Minimum	3.00		
	Maximum	4.00		
	Mean	3.8881	3.8524	3.9188
	SD	0.27250	0.23162	0.30911
Public relations	Minimum	3.00		
	Maximum	4.00		
	Mean	3.9299	3.9053	3.9520
	SD	0.19353	0.15368	0.23051
New media	Minimum	3.00		
	Maximum	4.00		
	Mean	3.9287	3.9053	3.9496
	SD	0.18547	0.15206	0.21534
Two-way communication	Minimum	2.67		
	Maximum	4.00		
	Mean	3.7651	3.7171	3.8118
	SD	0.39979	0.35094	0.44565
Media content management	Minimum	2.67		
	Maximum	4.00		
	Mean	3.8807	3.8462	3.9090
	SD	0.27086	0.23166	0.30807

Furthermore, the respondents also indicated that their public relations practices through new media follow two-way communication patterns. The relevant two-way communication, according to the respondents, is focused on obtaining audience feedback on certain media content. Their feedback is used to evaluate content and design new content accordingly (Froment et al., 2017). Finally, the respondents agreed that content management, being a strong consideration for their organization, is influenced by two-way communication. As also argued by Ahmed and Khan (2019), the audience as receivers of media content can evaluate well its quality and appropriateness. When dealing with media content, two-way communication can also help content designers and creators to think with more creativity and vigilance.

## 7 Implications and conclusions

This research has some implications. Firstly, it underlines the importance of building enhanced relationships between PR practitioners and media outlets. Trust and collaboration can be strengthened by fostering two-way interactions, resulting in more accurate and reliable media content. Furthermore, embracing the two-way communication model improves the accuracy of information shared in media content. PR professionals actively engage with journalists, providing accurate and timely information, reducing the chances of disseminating misinformation and rumors, and ensuring that the media has access to reliable sources and verified information. Furthermore, the two-way communication model allows effective crisis communication. PR practitioners can establish a collaborative relationship with media professionals, permitting the dissemination of accurate information and addressing concerns. Through open and transparent communication during times of crisis, organizations can control the narrative and minimize the spread of rumors or false information. Moreover, it is notable that implementing the two-way communication model contributes to public perception and trust. Organizations can build trust, credibility, and transparency by engaging in meaningful conversations with the public through the media (Michael, 2021). This fosters a positive perception among the public as they perceive the organization as responsive, open, and willing to engage in dialogue. Finally, the two-way communication model aligns well with the interactive nature of social media platforms. PR practitioners can integrate social media into their strategies, leveraging it to engage in conversations, receive feedback, and share information, therefore enabling broader engagement with the public, improving media content management, and encouraging two-way communication. Thus, it is concluded that managing media content is not a simple task (Froment et al., 2017). Audience and communication are two basic factors that play an important role in this regard. Furthermore, the role of public relations practices also enhances communication and content management practices, leading to even more constructive outcomes. Organizations that understand the importance of symmetrical communication readily adopt new media as providing an important communication pathway. As two-way communication provides access to audiences, it also means that determining and measuring their preferences is not a difficult task anymore. Moreover, online public relations practices contribute to the digital ecosystem and artificial intelligence by using modern technologies and digital means to improve communication and interaction with the public and customers. Artificial intelligence can help public relations teams analyze data and identify specific segments within their audience, improve the user experience, and provide appropriate digital services. It can also be used in crisis management and communicating with the public in emergencies through digital content management. Strategic collaboration between public relations and digital technology is vital to achieving success in the digital age. It means that public relations are based on bilateral communication and effective listening as it seeks to understand the needs and expectations of the public and interact with them continuously.

### 7.1 Limitations

This study has some primary limitations. First, this study only presents data from the employees of some selected media organizations, mainly privately owned, in the United Arab Emirates, which puts its applicability to state-owned media into question. Second, the study hypothesis also remained insignificant, which further limits the scope. Finally, the third limitation involves the convenience sampling technique, which has received much criticism due to the nature of its selection criteria. However, more studies, especially on state-owned media in the United Arab Emirates, can further highlight these significant findings. In particular, by using the proposed conceptual model, content management can be explored in the context of private media platforms.

## Data availability statement

The raw data supporting the conclusions of this article will be made available by the authors, without undue reservation.

## Ethics statement

The studies involving humans were approved by IRB-2023-199-Yarmouk University. The studies were conducted in accordance with the local legislation and institutional requirements. The participants provided their written informed consent to participate in this study.

## Author contributions

AA: Writing – review & editing, Writing – original draft. IM: Investigation, Writing – review & editing, Formal analysis. MA: Methodology, Writing – original draft. RA: Conceptualization, Writing – original draft. FA: Supervision, Visualization, Writing – original draft. MH: Project administration, Writing – review & editing.
